# Associations, Fees, Contracts, Etc.

**Published:** 1880-02

**Authors:** 


ASSOCIATIONS, FEES, CONTRACTS, ETC.—In all the professions and trades, associations or societies seem to be thought necessary, and have been and are being formed for mutual protection and the advancement of the interests of their members, pecuniary and otherwise. Such associations seem to dignify the callings of which they are the outcrop, and give to them and their members a better status in the communities in which they exist. People generally seem to regard individuals with more favor, *cæteris paribus*, who are known to be members of such organizations than they do those of the same occupation or profession who are not. This idea is not, as a rule, devoid of good foundation, as those who are correct in their feelings and actions naturally look for and expect similar conduct from their associates and contemporaries. But, unfortunately, all are not thus naturally inclined to honorable conduct and deportment, and this furnishes additional reason for the institution of societies in which such rules and regulations are adopted by the members as are necessary to secure concert of action on matters calculated to elevate and dignify the calling in the estimation of the community, and advance, or at least protect, the interest of the individual members. The below excerpt from a daily paper has "the ring of the *true* metal," as it shows how the lawyers of this city intend to hold such delinquents as might so far forget the duty they owe their confrères and a noble profession as to be guilty of a violation of its rules and regulations. The quotation is from the constitution of the Law Association, recently formed by the leading members of the bar of Baltimore:

" The constitution states that the object of the association is to elevate and dignify the profession and see to the proper administration of justice. Any member guilty of misconduct or malfeasance shall be expelled, and any member of the bar or judiciary, whether a member of the association or not, who shall be guilty of malfeasance shall be legally prosecuted, counsel to be employed for the purpose by the association on recommendation of the committee on grievances."

Now, ought not physicians to be actuated and governed by *as high* a sense of honor and justice to themselves and their constituents as lawyers? We contend that they should; and if so, what course should be pursued toward those who under-charge the " fee table " habitually when it is to their interest to do so, and who disregard the " code of ethics " by making annual contracts for professional attendance on individuals or families, sometimes for merely *nominal* sums, in order to retain the practice of certain families; and in less frequent instances covenant to attend certain parties or families *gratuitously*, even where they are able to pay a moderate bill, to secure the practice of relations or friends of larger means.

We open our columns to any who may wish to ventilate the conduct of such as are guilty of the unprofessional acts alluded to above, and when found here or elsewhere, to hold them up to the reprobation of the profession at large.

The below editorial, from a recent number of the Chicago *Medical Gazette*, is appropriate in this connection:

" *Medical Journalism.*—The profession has looked in vain for editorial criticism on the innumerable topics of medical interest. Occasionally an editorial appears on the code, on consultations, on the action of some health board, but independent, fearless, friendless and prolonged agitation on special medical subjects, such as form the essential part of political journalism, is almost unknown. The medical journalist should have both the energy and the hardihood to print anything that is right and that is in the interest of society, and if one printing prove insufficient, let him print again and yet again. If it be deemed prudent upon occasion to pour in the hot shot, hot and hissing, it is not for him to inquire whom they may hit. When expressions are to be made, let them be rendered, not in metaphysics nor in poetry, but in the cold facts. Let the editorial columns be many and full; let them bristle with independence, with liberality and with criticism; let them include the pith of the medical literature and of the medical interests of the world."

